# Structural Insight of Dopamine β-Hydroxylase, a Drug Target for Complex Traits, and Functional Significance of Exonic Single Nucleotide Polymorphisms

**DOI:** 10.1371/journal.pone.0026509

**Published:** 2011-10-20

**Authors:** Abhijeet Kapoor, Manish Shandilya, Suman Kundu

**Affiliations:** Department of Biochemistry, University of Delhi South Campus, New Delhi, India; University of South Florida College of Medicine, United States of America

## Abstract

**Background:**

Human dopamine β-hydroxylase (DBH) is an important therapeutic target for complex traits. Several single nucleotide polymorphisms (SNPs) have also been identified in DBH with potential adverse physiological effect. However, difficulty in obtaining diffractable crystals and lack of a suitable template for modeling the protein has ensured that neither crystallographic three-dimensional structure nor computational model for the enzyme is available to aid rational drug design, prediction of functional significance of SNPs or analytical protein engineering.

**Principal Findings:**

Adequate biochemical information regarding human DBH, structural coordinates for peptidylglycine alpha-hydroxylating monooxygenase and computational data from a partial model of rat DBH were used along with logical manual intervention in a novel way to build an *in silico* model of human DBH. The model provides structural insight into the active site, metal coordination, subunit interface, substrate recognition and inhibitor binding. It reveals that DOMON domain potentially promotes tetramerization, while substrate dopamine and a potential therapeutic inhibitor nepicastat are stabilized in the active site through multiple hydrogen bonding. Functional significance of several exonic SNPs could be described from a structural analysis of the model. The model confirms that SNP resulting in Ala318Ser or Leu317Pro mutation may not influence enzyme activity, while Gly482Arg might actually do so being in the proximity of the active site. Arg549Cys may cause abnormal oligomerization through non-native disulfide bond formation. Other SNPs like Glu181, Glu250, Lys239 and Asp290 could potentially inhibit tetramerization thus affecting function.

**Conclusions:**

The first three-dimensional model of full-length human DBH protein was obtained in a novel manner with a set of experimental data as guideline for consistency of *in silico* prediction. Preliminary physicochemical tests validated the model. The model confirms, rationalizes and provides structural basis for several biochemical data and claims testable hypotheses regarding function. It provides a reasonable template for drug design as well.

## Introduction

Human dopamine β-hydroxylase (DBH), a constituent of catecholamine biosynthetic pathway, catalyzes the conversion of dopamine to noradrenaline or norepinephrine [1]. The enzyme is expressed in noradrenergic nerve terminals of the central and peripheral nervous system, as well as in chromaffin cells of adrenal medulla. It is an important therapeutic target that has been associated to and implicated in several diseases and pathological conditions including Parkinson's, Huntington's chorea, hypertension, depression, cardiac heart failure, Tourette syndrome, etc. [2]–[5]. Inhibition of DBH may allow treatment of some of such disorders like hypertension and congestive heart failure [6]–[8]. DBH is inhibited by disulfiram, tropolone, etamicastat, nepicastat and several others. [8]–[11]. However, they often result in side effects or adversities and are frequently non-responsive to specific population and hence the search for new inhibitors with desired specificity and intensity is always on. Moreover, there has been no structural basis for understanding of substrate binding to human DBH that can help envisage better inhibitors. Reports of the success of inhibitors such as nepicastat [11] as potential drugs are not substantiated by analysis of their mechanism of binding to DBH that can help design of analogues or chemical modifications to enhance their efficacy.

On the other hand, a number of single-nucleotide polymorphisms (SNPs) have been identified for DBH [1], [4], [12]–[17]. However, their functional significance is largely unknown. There have also been contradictory reports regarding the influence of SNPs on enzyme activity. Thus, while Ishii et al. [18] reported that non-synonymous SNP resulting in A318S mutation alter enzyme activity, Li et al. [7] showed that the mutation do not influence enzyme activity at all. There has been no structural validation, either way, for such contrasting results. In addition, functional significance of domains of DBH other than the ones containing the active site has not yet been elucidated.

A primary requisite for rational drug design, *in silico* inhibitor screening, understanding functional significance of SNPs and domains in DBH is a three dimensional structure of the enzyme. As of date, no crystal structure is reported for the enzyme (www.pdb.org) resulting in lack of global structural insight, though wealth of biochemical data and *in silico* studies of the active site domain are available for DBH [19]–[24].

The use of biochemical knowledge with regard to DBH for a structural insight was contemplated. DBH is a colorless monooxygenase containing a total of eight disulfide bonds [25]. The active unit of the enzyme is a tetramer of molecular weight 290000 Da, formed by non-covalent interactions between two dimers held together by two interchain disulfide linkages [19], [26], [27]. The enzymatic reaction is known to proceed through redox reaction in which the two Cu (II) centers of the resting enzyme are first reduced by ascorbate to an active Cu (I) state [21], [28]. Active site structure has been probed by EPR spectroscopy and other methods to obtain information on metal-binding amino acid residues and the coordination and geometry of the two copper atoms [20], [21], [29]–[35]. PHM (Peptidylglycine alpha-hydroxylating monooxygenase; 1PHM), with a 27% sequence identity to DBH, was shown to be the evolutionary precursor of the enzyme [36]. The three-dimensional coordinates and mechanistic data for the chemical and electron transfer steps in catalysis of PHM were thus used to model a partial structure of rat DBH to show the position of the catalytically important residues in the enzyme [28]. However, the model displayed Cu binding domains only and a structure for full-length protein is still lacking. The difficulties in crystallizing the protein have compounded the problem.

We hypothesized that combination of biochemical information, computational data, and sequences of DBH available from multiple species can be used to map a three-dimensional structure of the human counterpart of the enzyme *in silico*. The idea was to use a subset of the available biochemical information as guidelines to build a model that can be subsequently validated by observing whether the structure successfully corroborates the other subset of the biochemical data. In the current investigation, an attempt has been made to model the complete structure of human DBH with experimental evidences as guiding principles for computational framework. The model has been built into a tetramer revealing domain organization, oligomerization and active site details. Also, the locations of SNPs thought to be important for activity have been identified and certain controversial results analyzed. Substrate and inhibitor docking in the active site have also been inspected. This is the first report of structural details of human DBH obtained *in silico* and will expedite better understanding of the structure-function relationship of the enzyme, functional significance of SNPs and rational design of potentially important therapeutics. The novel approach of building the model using biochemical information in conjunction with other commonly used techniques is an important step towards solving protein structures in general.

## Results and Discussion

### Primary Structure Analysis

The human DBH sequence from NCBI database, with accession number P09172, was targeted for investigation. Protein sequence BLAST against non-redundant database in NCBI showed that DBH share similarity with peptidylglycine alpha-hydroxylating monooxygenase (PHM) with 27% sequence identity. PHM was shown to be the evolutionary precursor of DBH [36]. Bhaduri et al. [37] analyzed DBH sequences from twelve organisms, which were revisited for the sake of current investigation (Table S1). Human DBH sequence shares maximum identity (86%) with *Sus scrofa* (pig). Significant similarity was also obtained with other mammalian species indicating the significance of this protein in general.

**Figure 1:**
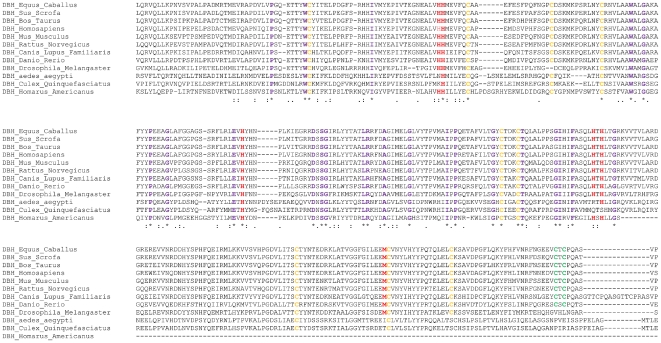
Multiple sequence alignment of DBH sequences. Eleven DBH sequences (horse, pig, cattle, mouse, rat, dog, zebra fish, drosophila, *Aedes aegypti*, mosquito, lobster) were aligned to the human DBH sequence [37]. Residues that are identical are highlighted in purple font; Cys residues involved in intramolecular disulfide bonds are highlighted in yellow color while the Cys residues involved in interchain disulfide links are colored green. Copper binding sites are highlighted in red color.

Amino acid residues absolutely conserved in DBH proteins were identified from a multiple sequence alignment shown in Figure 1 and partly in Bhaduri et al. [37]. Only 45 of the total residues are completely conserved when species as diverse as the *Drosophila* and *Homarus* (lobster) are included in the comparison. However, 350 of the total residues were completely conserved when only species with significant alignment were used for comparison. All conserved residues are highlighted in purple color. The five His residues and one Met residue that could be important for interaction with the active site Cu ions, by analogy to PHM [29], [37], are highly conserved except in case of A*edes aegypti* and *Culex quinquefasciatus* where one of the His is replaced by Asn and Lys, respectively. The His and Met residues that putatively interact with the other copper centre, by comparison to PHM [28], are also replaced in both the above organisms. Fourteen Cys residues that possibly form structurally important disulfide bonds, as evidenced by biochemical investigation [25], are highly conserved. However, in case of *Drosophila*, *Aedes*, *Homarus* and *Culex*, the latter two Cys residues are absent indicating that interchain disulfides are probably absent in the corresponding proteins. This signifies that in lower organisms, DBH might not undergo oligomerization and may be functional in monomeric form. Prigge et al. [28] demonstrated the catalytically important residues in rat DBH based on their homology with PHM (PDB ID: 1PHM). It was shown that Glu268, Glu369 and Tyr494 form complex hydrogen bonding network with substrate. These residues are highly conserved (highlighted in light blue) except in case of *Aedes* and *Culex* where none of the three residues are present. Due to such differences with DBH of lower organisms, our investigation will be restricted to comparisons of human DBH to mammalian DBH only, whenever required.

**Figure 2:**
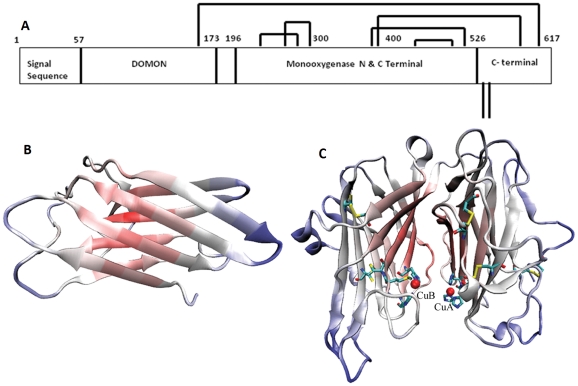
Domain organization of human DBH sequence. *A.* Domain architecture of human DBH obtained using CDART. Shown are the three domains: DOMON domain (sequence position 57–173), Cu type II monooxygenase N-terminal domain (sequence position 196–344), and Cu type II monooxygenase C-terminal domain (sequence position 359–526). Intramolecular disulfide bridges are shown by closed lines while the two open lines correspond to cysteine residues involved in interchain disulfide bridges. *B.* Structure of DOMON domain modeled using I-TASSER server. *C.* Structure of the two Cu type II monooxygenase N- and C-terminal domains based on reduced peptidylglycine alpha-hydroxylating enzyme (PDB ID: 1PHM). The two Cu atoms are represented as red spheres. Residues forming disulfide links are colored by atom type (carbon is cyan, nitrogen is blue, oxygen is red and sulfur is yellow).

Primary structure of human DBH is 617 amino acids in length with 15 cysteine residues of which 14 are involved in disulfide bond formation [25]. Cys154-Cys596, Cys232-Cys283, Cys269-Cys295, Cys390-Cys503, Cys394-Cys565 and Cys466-488 are involved in intramolecular disulfide bonds whereas two cysteines, at positions 528 and 530, are involved in intermolecular disulfide bonds (Figure 2a). The location of these residues and disulfides served as milestones in model building, frequently guiding the orientation of peptide backbone, proximity of amino acid side chains and subunit interaction, especially since all intrasubunit disulfides are conserved across species (Figure 1). DBH contains a total of 64 negatively charged residues (Asp + Glu) and 52 positively charged residues (Arg + Lys) giving protein an overall charge of −12. Since charged residues in globular proteins mostly reside on surfaces, they also provide guidelines for protein model building.

Search for domain architecture of DBH using CDART [38] shows that DBH consists of three major domains as shown in Figure 2a, mentioned in Table S1 and also reported earlier [37]. The first major domain on the N-terminal side is DOMON domain [37], [39], which is located from position 57 to 173. Preceding this is a signal sequence. Following the DOMON domain is Copper type II ascorbate-dependent monooxygenase N-terminal domain located from position 196 to 344 followed by Copper type II ascorbate-dependent monooxygenase C-terminal domain located from position 359 to 526 [37]. Between the DOMON domain and the monooxygenase domains is a short stretch of amino acids as well. The sequence also showed a C-terminal tail without sequence homology to any known domain. There are about 182 more sequences reported in NCBI with similar domain architecture consisting of one DOMON domain followed by two monooxygenase domains.

The DOMON domain has been identified in several secreted and transmembrane proteins from both plants and animals and is about 110–125 residues in length [39]. It is found in 1–4 copies and in association with other domains such as Cu ascorbate-dependent monooxygenase, Reelin domain, SEA domain, trypsin inhibitor-like domain (TIL) and epidermal growth factor domain. Structure for DOMON domain is not available; however, it has been proposed to form a β-sandwich structure with 7–8 core strands [37], [39] supported by a buried core of conserved hydrophobic residues. Human DBH DOMON domain modeled using I-Tasser conforms to this hypothesis and shows a beta-sandwich structure (Figure 2b). This domain may function as a module in mediating a range of extracellular adhesive interactions and might also be needed for the non-covalent tetramerization of DBH or for interaction between DBH and specific proteins [37], [39]. In general, DOMON domain has been shown to be involved in heme and sugar recognition [40]. Human DBH showed two glycosylation sites, one at position 64 (present within the DOMON domain) and the other at 184 (located close to this domain) [41].

The two monooxygenase domains form the catalytic unit of the enzyme and each one binds to one Cu atom required for the activity of the enzyme. A crystal structure for this domain from PHM is known [28]. In the enzymes of the PHM family, N-terminal domains are approximately 140 residues long and their structural unit consists of a β-sandwich of two antiparallel β-sheets composed of approximately ten β-strands. There are three disulfide links connecting the strands and loops as seen in rat PHM (PDB ID: 1PHM; Figure 2c); however in case of bovine DBH this domain contains only two disulfide links and the third link is missing as there is no Cys residue at the corresponding position [25]. This domain binds one Cu atom designated as CuA, which is believed to be involved in ascorbate binding. The C-terminal domain is approximately 160 residues long and its structural unit also consist of a β-sandwich comprising two antiparallel β-sheets composed of approximately twelve β-strands and one 3_10_ helix. Though this domain consists of two disulfide links in the enzymes of PHM family (Figure 2c) but in case of bovine DBH it contains three disulfide links [25]. It also binds one Cu atom designated as CuB, which is supposed to be the site for substrate binding and hydroxylation. Human DBH, as evident from sequence comparison, should exhibit many of these characteristics in its three-dimensional model.

### Secondary Structure Analysis

**Figure 3:**
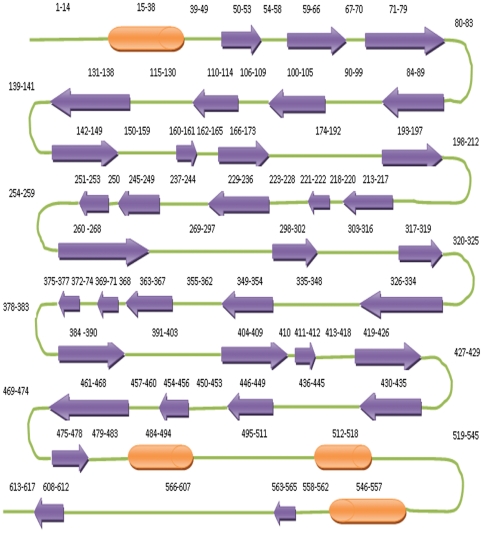
Secondary structure distribution of human DBH sequence obtained from PredictProtein server. Purple arrows indicate β-strand; orange cylinders indicate α-helix while loops are depicted as green lines. The numbers indicate the position within the sequence.

The secondary structure prediction of human DBH, performed using PredictProtein tool, classified the overall protein as “Mixed” with 61% % of total amino acids being in the form of loops. The high content of loops in the protein architecture with putative flexible structure might prevent formation of diffractable crystals. The predicted β-sheet and α-helical contents were 30% and 9%, respectively, as represented schematically in Figure 3. The prediction matches well with structural data obtained for individual domains from other sources like PHM (Figure 2c) or models of individual domains of human DBH like DOMON domain (Figure 2b). So secondary structural information can be relied upon for building model whenever ambiguity arises or restrictions are required to assign conformation in three-dimension. Further, primary sequence analysis and secondary structure predictions are more robust than tertiary structure predictions and hence the former were given priority over the later whenever contradictions arose in the current investigation.

### Model Building and Validation – Three-dimensional structure

**Figure 4:**
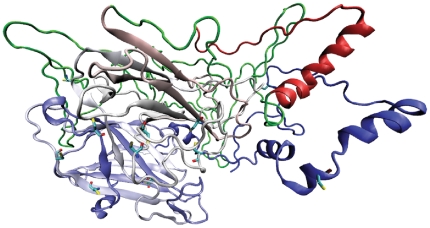
Model of human DBH obtained using I-TASSER server. The cysteine residues are colored by atom type (carbon is cyan, nitrogen is blue, oxygen is red and sulfur is yellow). Region of structure from 39–209 is colored green.

Modeling DBH protein structure using computational techniques such as homology modeling and threading was not feasible as there are no structural homologues available for full-length sequence of the enzyme. PHM is the only enzyme that exhibits homology to the N- and C-terminal monooxygenase catalytic domains of DBH. Attempt was made to model DBH using *ab-initio* approach (Figure 4). However, the model was not reliable as none of the disulfide linkages reported experimentally [25] were observed in the model. The cysteine residues were not in proximity for formation of covalent linkage indicating that the backbone conformation was erroneous. Moreover, the region from 39–209 (region containing DOMON domain; represented in green in Figure 4) was modeled as loop. This contradicts secondary structure prediction depicted in Figure 3 as well as the structural organization obtained for human DOMON domain when modeled individually (Figure 2b) or as proposed for such domains in other proteins like PHM [39]. The model was thus unacceptable.

**Figure 5:**
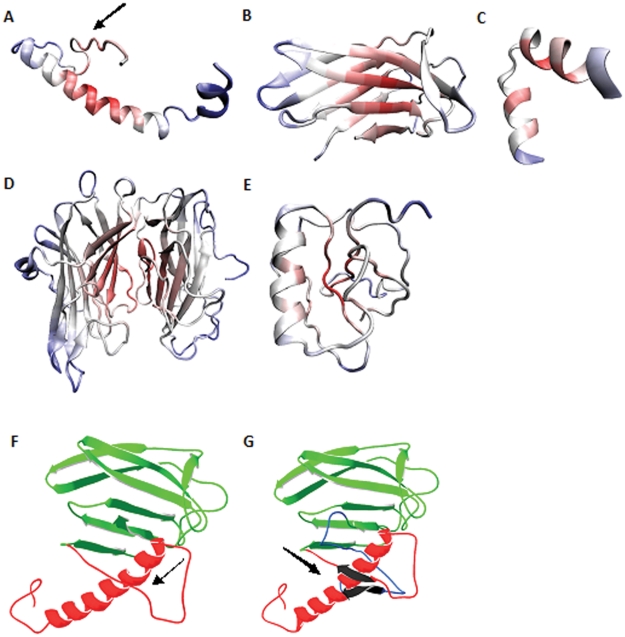
Structure of DBH protein fragments modeled using I-TASSER and EsyPred3D. *A.* Fragment 1 spanning the sequence from 1–56. *B.* Fragment 2, DOMON domain, sequence spanning from 57–173. *C.* Fragment 3, the connecting loop between the DOMON domain and the monooxygenase domain, spanning the sequence from 174–195. *D.* Fragment 4, the N-and C-terminal monooxygenase domains, spanning the sequence from 196–526. *E.* Fragment 5, the C-terminal region. *F* and *G* depict the orientation of fragment 3. *F.* Modeled structure from sequence position 1–173; with region 1–56 highlighted in red and DOMON domain (57–173) colored green. No β-strand is seen in the region 51–53 as indicated by arrow. *G.* Modeled structure from sequence position 1–197; with region 1–56 highlighted in red and DOMON domain (57–173) shown in green; fragment 3 depicted in blue. β-sheet is shown in black color between 51–53 and 193–197.

The problem was subsequently overcome by modeling smaller fragments of proteins as mentioned in the methods section. I-TASSER (*ab-initio* method) modeled the individual fragments in agreement with the secondary structure predictions (Figures 5a–5e) except fragment 3 (amino acid sequence 174–195), which was modeled as helical structure but is predicted to be loop (Figure 5c).

**Figure 6:**
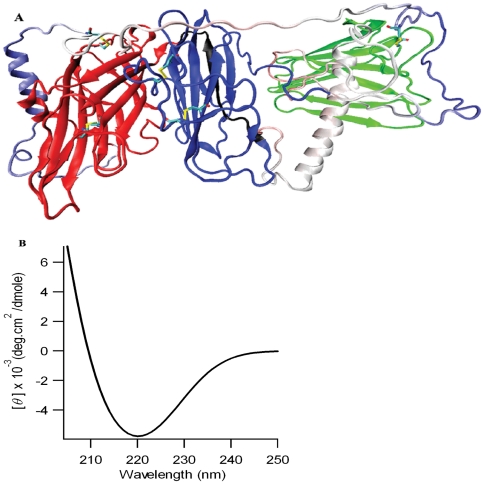
Complete model of human DBH enzyme and its experimental validation. (A) Residues forming disulfide links in the model are colored by atom type (carbon is cyan, nitrogen is blue, oxygen is red and sulfur is yellow). (B) Circular dichroism of DBH enzyme predicted from the model. The predicted spectra matches well with experimental circular dichroism spectrum of bovine DBH [44] .

The basic idea was then to assemble the *ab-initio* modeled fragments around the homology modeled fragment 4, the N- and C-terminal monooxygenase catalytic domain. The complete model was successfully assembled by reproducing the torsion angles of the individual fragments to the extended polypeptide chain and changing the angles wherever necessary to fulfill geometric requirements. Orientation of fragment 3 (residues 174–195; Figure 5c), the loop region between the DOMON domain and the N-terminal monooxygenase domain, was important as the orientation of this loop decides where rest of the structure goes. Loop database present within Swiss-PDB Viewer was tried to predict the structure of this region (174–195) but the predictions could not orient the loop properly. The clue to orient this loop region was obtained from the secondary structure map (Figure 3). There is a β-sheet predicted in the regions 50–53 and 193–197. Interestingly, there is no β-sheet in the region 50–53 in the absence of the rest of the structure as in seen in the model for fragment 1, indicated by arrow in Figure 5a and in Figure 5f. Also, all other predicted β-strands belong to one or other of the three domains except the strand predicted between 193–197. So, it was argued that these two strand regions must interact to form a β-sheet in the DBH molecule. Many different loop orientations were then tried by manually adjusting the torsion values to bring these strands together. However, the sheet was seen in only one particular orientation depicted in Figure 5g and was thus acceptable. The N-terminal structure (sequence from 1–197) generated was then assembled with the 4^th^ fragment. The homology modeled 4th fragment contained only two disulfide links between Cys232-Cys283 and Cys466-Cys488. However, it has been reported that the two domains together are involved in five disulfide linkages between Cys232-Cys283, Cys269-Cys295, Cys390-Cys503, Cys394-Cys565 and Cys466-Cys488 [25].Conforming to experimental results, the structure was changed locally to introduce disulfide link between Cys269-Cys295. Assembly of 5^th^ fragment was based on introducing two disulfide linkages between Cys394-Cys565 and Cys154-Cys596. Thus, all reported disulfide linkages were modeled except the one between Cys390-Cys503. Introduction of this particular disulfide link required lot of changes in the domain structure, which resulted in major distortion of the overall configuration of the C-terminal domain. So it was left undone. Literature search showed that there could be erroneous determination of the number of disulfide bridges in solution for proteins, which were later rectified in three dimensional structures of those proteins [42]. The Cys390-Cys503 disulfide bond could be an experimental aberration as well and the final verification will have to await experimental three dimensional structure of the enzyme. Figure 6a shows the complete model of human DBH generated with all disulfide links highlighted. DOMON domain, Cu type II N-terminal domain and Cu type II C-terminal domains are highlighted in green, blue and red color respectively. Quality of the overall structure was estimated by calculating the Ramachandran plot. Approximately 91% residues lies in allowed region (favored + allowed regions) and rest of them are outliers. However, this can be expected as 60% of structure is predicted to be loop and Ramachandran plot does not define values for the residues in loop regions. It can be seen from Table S2 that almost all the outlier residues belong to loop region. Thus, DBH was modeled using experimental information in conjunction with computational techniques and manual intervention.

### Active Site Structure - Introducing two Cu atoms

**Figure 7:**
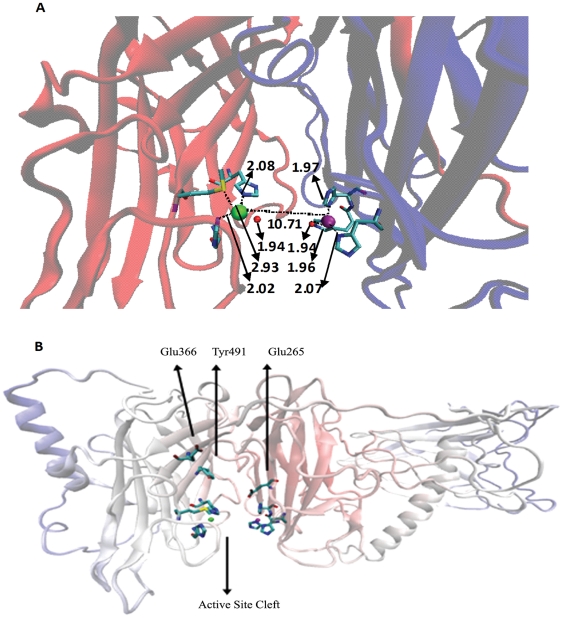
Active site architecture of DBH. *A.* Distance between the metal binding sites and Cu atoms are highlighted. Cu_A_ is depicted as purple sphere; Cu_B_ is depicted as green sphere. Two water molecules are represented as red spheres. Metal binding residues are colored by atom type (carbon is cyan, nitrogen is blue, oxygen is red and sulfur is yellow). *B.* Active site cleft along with the active site residues highlighted. Also, shown are Glu265, Glu366 and Tyr491, residues that play important role in catalysis. Residues are colored by atom type (carbon is cyan, nitrogen is blue, oxygen is red and sulfur is yellow).

EPR and EXAFS studies have shown the coordination for two Cu atoms in the oxidized state to be Cu_A_(His)_3_(H_2_O) and Cu_B_(His)_2_X(H_2_O), where X is either His or O-donor ligand or solvent [21]. The imidazole groups are located at 1.99 Å from Cu atom whereas the O-donor groups reside at an average distance of 1.94 Å. Using these data and homology with PHM (PDB-ID: 1PHM), the two Cu atoms were introduced in the modeled structure as shown in Figure 7a. At the Cu_A_ binding site (purple sphere), His262, His263 and His333 interact with Cu atom through their delta nitrogen atom (ND1) and are present at 1.96 Å, 1.97 Å, and 2.07 Å, respectively, from Cu center. These distances are similar to those reported by EPR investigation. Similarly, at the Cu_B_ binding site (green sphere), His412 and His414 interact with Cu atom through their epsilon nitrogen atom (NE2), and are present at 2.08 Å and 2.02 Å respectively, from Cu center. These distances in the model are also in reasonable agreement with those observed from experiments as mentioned above thus providing confidence in the model. X, whose ligand identity is unknown, was estimated to be located at 2.56 Å from Cu atom through biochemical studies [24]. It is impossible to identify what X is in an *in silico* model and would await final confirmation only from X-ray crystallographic studies. We did not observe any (O/N) amino acid ligand at 2.5–3 Å in our model. The possibility remains that the ligand is a solvent molecule. However, both His439 and Gln486 are likely candidates being located at ∼6 Å away from Cu_B_, provided the ligand X is contributed by the polypeptide. It is interesting to note, that both His439 and Gln486 are highly conserved in primary structure, which argues for a very special role for these two amino acid residues. Any requisite conformational change could bring these two residues within the distance observed experimentally. Two water molecules, shown as red spheres in Figure 7a were also introduced at the two Cu sites at 1.94 Å distance from Cu.

It has been reported, however, that in the reduced state of the enzyme, there are dramatic changes in DBH protein structure [21]–[24]. The two water molecules mentioned above are lost and the Cu atoms are present in reduced coordination state (less than 4). The model presented here can serve as a starting point for simulation of conformational changes to mimic the reduced state of the enzyme. In such a state, Cu_B_ coordinates with a S atom from Met487 present at 2.23 Å [24]. In the oxidized state in our model, Met487 does not coordinate with Cu atom and is present at 2.93 Å in the structure (Figure 7a). Such strategic and proximal placement of Met487 does indicate that on conformational change typical of reduced state the S atom could move closer to the Cu_B_ atom, thus corroborating experimental finding. Such a movement can eliminate water away from active site pocket and reduce coordination state of Cu below 4. Despite the requirement for two Cu centers per catalytic unit, there is no evidence for short-range magnetic interaction and hence each Cu (II) center is believed to be mononuclear. A lower limit of 7 Å is defined between the two Cu centres [31] and the proposed model shows that indeed the Cu sites are 10.27 Å apart. Between the two Cu sites is a typical cleft that is fully accessible to solvent, which probably links the two Cu sites. Figure 7b highlights the active site cleft with the two Cu centers along with their metal binding residues and the residues Glu265, Glu366 and Tyr491 that are suggested to play important role in the catalysis of the enzyme [28].

### Quaternary Structure

**Figure 8:**
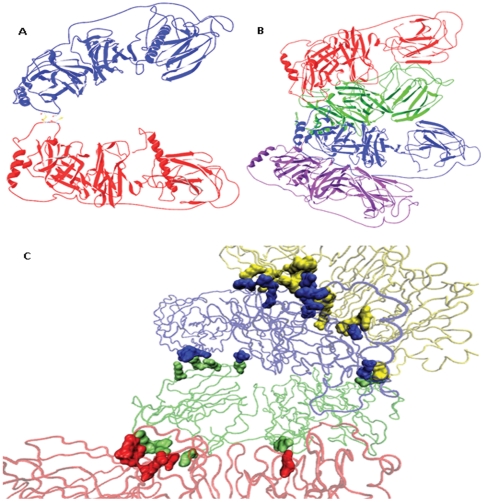
Models for oligomer structures of DBH. *A.* Model for the dimeric form of DBH. Chain A and B are shown in blue and red, respectively. *B.* Model for the tetrameric form of the enzyme. Chain A, B, C, and D are depicted in blue, red, green and purple respectively. *C.* Residues forming tetramer interface. Subunits are highlighted in tube conformation with chains A, B, C, and D highlighted in blue, red, green and yellow, respectively. Residues are represented in SURF conformation with colors same as that of corresponding chain color.

It has been shown that the two subunits form a dimer connected through interchain disulfide link between Cys528A-Cys528B and Cys530A-Cys530B [25]. Figure 8a shows the probable dimer structure generated by rotating and translating the subunits, for optimum geometry, linked through the two disulfide bonds mentioned above. The dimers are held through non-covalent interaction to form the tetramer, which is known to be the active unit of the enzyme [19]. Figure 8b shows the probable tetramer structure for the soluble form of the enzyme in which each subunit lacks the signal peptide. The subunits are not very intricately woven, but seem to be adjacent and planar. This presents the first oligomeric structure for DBH. Figure 8c highlights the residues involved in interface formation and provides an image of the subunit interface. Table 1 lists the residues involved in interface formation. Different non-bonded interactions like hydrophobic contacts, electrostatic interactions, interchain H-bonds and salt bridges were inspected for interface identification. Almost all the residues involved in interface formation are conserved across the species showing significant alignment implying that the tetramer structure adopt almost similar fold and is significant to the physiological function of the enzyme. It is obvious that the DOMON domain can potentially promote tetramerization of the subunits. The DOMON domain alone contributes almost 26% of the interface amino acids. On the other hand, the C-terminal domain could be important for dimerization of the enzyme since the two disulfides that force interchain linkage are both present in the C-terminal domain. The flexibility in this region could facilitate the subunit recognition and interaction.

**Table 1:** Residues involved in tetramer interface formed by different non-bonded interactions.

Interchain H-bonds	Salt Bridge	Electrostatic Interactions	Hydrophobic Interactions
Glu576A–Thr254D	Glu381A–Lys451D	Chain A Thr139,Pro140,Glu141, Gly142,Phe241,Val253, Lys286,Arg291,Pro322, Gly323,Arg326,Val370, Tyr371,Pro373,Val374, Pro379,Arg380,Val478, Gln574,Gly575,Glu576, Trp577,Leu579	Chain A Glu141,Phe241,Arg291, Pro322,Val374,Arg380, Arg572,Gln574,Gly575, Trp577
Arg326A–Glu60D	Arg326A–Glu60D	Chain B Leu77,Arg79,Pro140, Lys451	Chain B Glu60,Leu77,Arg79, Pro140
	Lys567A–Glu141D	Chain C Gln135,Gln137,Pro140, Lys148,Met190,Ile340, Val527,Cys528,Gly614, Lys616	Chain C Gln137,Met190,Val527, Cys528,Ile612,Gly614, Lys616
	Arg572A–Glu141D	Chain D Asp54,Pro55,Glu56, Gly57,Glu60,Gln75, Leu77,Arg80,Glu141, Ile252,Val253,Thr254, Glu258,Gly305,Lys307, Phe309,Val527,Cys528	Chain D Pro55,Glu56,Gly57, Gln75,Leu77,Glu141, Ser179,Val253,Phe309
	Arg291A–Glu258D		
	Glu141B–Lys616C		

Salt bridge identification was performed with cut off distance of 7 Å. Electrostatic interactions were determined within 4 Å distance.

### Physicochemical validity of the model

A preliminary physicochemical test of the validity of the model was achieved by comparing the experimental partial specific volume of DBH with the one calculated using soluble form of the computed model. The predicted values of partial specific volume are 0.758 ml/g (with volume = 8.17589e-20 ml calculated using ^3^V server) and 0.770 ml/g (with volume = 8.3126e-20 ml calculated using VADAR server) which are comparable to the experimentally known value for bovine DBH (0.731 ml/g) and human pheochromocytoma DBH (0.72 ml/g) [43]. A further confirmatory test involved comparison of experimental and calculated circular dichroism (CD) spectrum of DBH. The CD spectrum predicted from DBH model is shown in Figure 6B. The calculated spectrum is comparable in both overall shape (with a single negative peak at 219 nm) and maximum ellipticity value (approximately −5900 deg.cm^2^/dmole) to the experimental spectrum for bovine DBH (single negative peak at 215 nm and mamimum ellipticity value of approximately −6000 deg.cm^2^/dmole) reported previously [44]. Such tests prove that the *in silico* model proposed in the present investigation is valid. The minor differences in negative wavelength maximum for predicted CD spectrum is acceptable and statistically not significant since the prediction algorithm is not perfect (as seen when known crystal structures of model proteins failed to reproduce the experimental CD spectrum to perfection; figures not shown). Moreover, no CD spectrum for human DBH is yet known, and the comparison performed here was with bovine DBH, whose experimental CD spectrum might have differences from its human homologue, resulting in the observed differences in the predicted spectrum.

### Single Nucleotide Polymorphism

**Figure 9:**
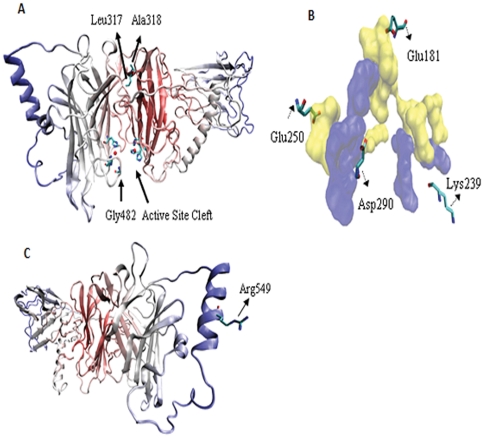
Mapping SNPs on the DBH structure. *A.* Shown are the position of SNPs Leu317, Ala318 and Gly482. The two Cu atoms are represented as red spheres. *B.* Shown are the position of SNPs Glu181, Glu250, Lys239 and Asp290 located near the interface of chain A and D. Interface is shown as SURF conformation with chain A colored blue and chain D in yellow. *C.* Arg549 is exposed to surface. Its substitution to Cys may result in disulfide-based oligomerization. Residues are colored by atom type (carbon is cyan, nitrogen is blue, oxygen is red and sulfur is yellow).

A number of SNPs are known to exist within coding regions those results in synonymous changes in the amino acid sequence of the enzyme [12], [45]. Identifying the location of these SNPs within the structure of the enzyme and their interactions with neighboring amino acid residues is critical in determining their functional significance. Knowledge of the effect of these SNPs on the activity of the enzyme can help with rational drug design. Eighteen of such SNPs were studied as listed in Table 2 along with their neighboring residues within 6 Å radius of the concerned SNP. Tetramer is known to be the active form of DBH whereas no activity is reported in the dimer or monomer form. Hence, SNPs close to active site region or the ones present in the (or near the) interface region of the tetramer may have important implications on the activity of the enzyme. Change in amino acid at position 318 from Ala to Ser was reported to have no effect on the activity of the enzyme [7], while another group [18] reported the contrary. Ala318, as evident from Figure 9a, is present in a region distant from the copper binding sites, opposite to the active site cleft and thus may not have any effect on the activity. This finding confirms the first report and provides a structural basis to the contradictory results. Similarly, the Leu317→Pro317 substitution is not expected to affect enzyme activity. However, substitution of Gly to Arg at position 482 may have subsequent effect on the activity of the enzyme as it is present close to the active site, right at the opening of the cleft (Figure 9a). As can be seen from Table 2, active site residues His414 and Met487 are present within 6 Å radius of this residue and could indirectly influence DBH function as well. Other SNPs that might have functional significance are Glu181→ Gln181, Lys239→Asn239, Glu250→ Gln250, and Asp290→Asn290 as they are located near the interface of the tetramer as shown in Figure 9b. Substitution of Arg549 with Cys might also have functional significance as the Cys is surface exposed and might be involved in random interchain disulfide links resulting in non-native dimerization [13] (Figure 9c). Other SNPs listed in Table 2 do not seem to be as important for the activity of the enzyme, as neither are they close to active site region nor to the tetramer interface.

**Table 2:** SNPs causing synonymous changes in protein and neighbouring residues within 6 Å radius.

SNPs	Residues in 6 Å radius
Gly12→Ser12	Ser9,Leu10,Pro11,Gly12,Pro13,Ser14,Met15,Arg16
Arg16→Trp16	Pro11,Gly12,Pro13,Ser14,Met15,Glu17,Ala18,Ala19,Phe20
Gly88→Ala88	Leu86,Phe87,Met89,Ser90,Gly93,Glu94,Leu95,Ala98,Asp99,Leu100,Val101, Leu168,Val169,Tyr170
Asp106→Ala106	Trp104,Thr105,Gly107,Asp108,Thr109,Arg138
Glu181→Gln181	Phe29,Ile32,Leu33,Pro52,Leu53,Asp54,Lys82,Ile172,Leu173,Ser179,Leu180, Ala182,Pro200
Asn201→Ser201	Phe177,Arg178,Leu198,Lys199,Pro200,Ile202,Pro203
Ala211→Thr211	Leu207,Pro208,Ser209,Asp210,Ala211,Cys212,Thr213,Tyr353,Thr354, Ala355
Lys239→Asn239	Leu237,Pro238,Gly240,Phe241,Ser242,His244
Glu250→Gln250	Lys248,Tyr249,Pro251,Ile252,Ile349,Arg350,Tyr352
Asp284→Asn284	Pro282,Cys283,Ser285,Lys286,Phe481
Asp290→Asn290	Pro271,Lys288,Pro289,Arg291,Leu292,Gln574,Gly575,Glu576
Leu317→Pro317	His245,Ile246,Tyr249,Gln268,Ala315,Gly316,Ala318,Phe319,Asn361, Ala362
Ala318→Ser318	Arg243,His244,His245,Ile246,Gly316,Leu317,Phe319,Gly320,Ser324, Asn361
Asp460→Asn460	Asp392,Leu424,Val425,Arg426,Val456,His457,Pro458,Gly459,Val461, Leu462
Thr467→Met467	Phe384,Ile385,Leu386,Gly417,Arg418,Lys419,Ser465,Cys466,Tyr468, Asn469
Gly482→Arg482	His414,Gly480,Phe481,Ile483,Leu484,Glu485,Met487
Trp544→Ser544	Trp431,His517,Leu518,Val542,Pro543,Asn545,Ser546, Phe547,Asn548
Arg549→Cys549	Asp427,Gly428,Arg429,Glu430,Leu518,Pro543,Trp544,Asn545,Ser546, Phe547,Asn548,Arg549,Asp550,Val551, Leu552,Lys553,Ala554,Tyr556

### Analysis of substrate binding

**Figure 10:**
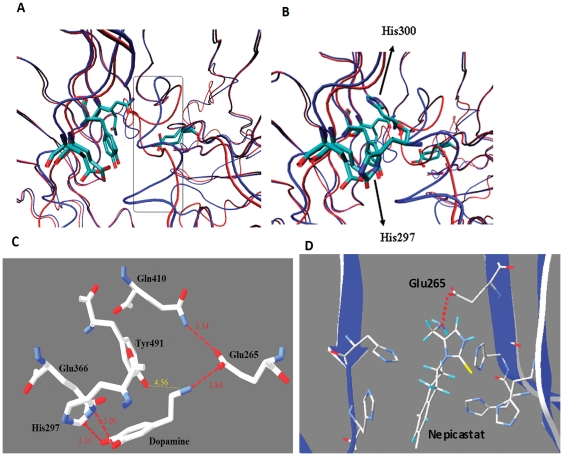
Residues important for catalysis. *A.* Active sites for rat DBH and human DBH superposed. Difference in the loop orientation located near the active site region is highlighted. Shown are the residues Glu265, Glu366, Gln410 and Tyr491. Both the models are depicted in tube conformation with rat DBH shown in red and human DBH in blue. Metal binding residues are colored by atom type (carbon is cyan, nitrogen is blue, oxygen is red). *B.* Difference in the orientations of active site residue His300 from rat DBH and His297 from human DBH. *C.* Residues involved in hydrogen bonding with the substrate dopamine. The hydrogen bonds are highlighted in light pink along with their distances. The distance between Tyr491 and dopamine is highlighted in yellow and is too long to form proper hydrogen bond. *D.* A potential drug, nepicastat docked to human DBH. Nepicastat is shown sandwiched between the two Cu centers and the highlighted His residues. The inhibitor hydrogen bonds to Glu265.

DBH shares 27% sequence similarity with PHM. Positions corresponding to five His residues and one Met residue involved in metal binding are very well conserved. The position of Cys residues forming disulfide links is conserved as well. The structure and mechanistic data of PHM were thus extended to the monoxygenase domain of rat DBH (modeled using the structure of rat PHM as a template) to display the position of the catalytically important residues in DBH [28]. In the rat DBH model, residues Glu265, Glu366, Gln410 and Tyr491 (numbering based on Human DBH sequence) were shown to form H-bonding network with dopamine that stabilized the substrate interaction. However, the interaction of Tyr491 with dopamine was dubious as substitution of this Tyr with Phe through site-directed mutagenesis in rat PHM yields active enzyme with little effect on its activity [28]. Figure 10a highlights the rat DBH (in red; modeled using 1PHM as template) and modeled human DBH structures along with the four residues (Glu265, Glu366, Gln410 and Tyr491) thought to be important in catalysis. The two superposed structures show a marked deviation in the orientation of the loop (encircled in Figure 10a) located close to the active site region. As a result His297 is located close to the Glu366 residue in human DBH and restricts the dopamine hydroxyl group from facing upward. In contrast, the corresponding His residue (His300) in rat DBH faces away from Glu366 as shown in Figure 10b. Hence it is essential to investigate human DBH in details without relying solely on rat DBH. Figure 10c highlights the interaction of the enzyme with the substrate in the structure of human DBH/dopamine complex where dopamine was docked manually. Similar hydrogen bonding network can be seen as demonstrated by Prigge et al. [28] for rat DBH. However, remarkably, no hydrogen bond was observed between Tyr491 and dopamine and the distance between them is 4.56 Å, large for H-bond to form (Figure 10c). This emphatically explains why no change in enzyme activity was observed experimentally when Tyr491 was mutated [28], as mentioned above. This signifies and strongly validates the model since it can faithfully corroborate experimental findings. Furthermore, the model shows that His297 contributes immensely in stabilizing the dopamine by forming a hydrogen bond as shown in Figure 10c. His297 is also well conserved across the species and our finding provides a structural rationale for such conservation.

### Mode of Inhibitor Binding

Diseases such as congestive heart failure can be treated by DBH inhibition, which directly modulates sympathetic nerve function [6]. One such novel, selective and potent drug identified for inhibition of DBH is nepicastat [11]. Preclinical studies have shown that nepicastat produces gradual modulation of sympathetic nervous system by inhibiting the biosynthesis of noradrenaline [6]. However, lack of proper three-dimensional structure of DBH has precluded structural investigation of the complex between the enzyme and the inhibitor and the understanding of the mechanism of inhibition. In order to gain insight into the structural aspect of inhibition, nepicastat was docked to the soluble form of DBH model. Out of several putative complexes obtained, we inspected one where nepicastat docked in the active site cleft with acceptable energy parameters. As shown in Figure 10d, nepicastat is sandwiched between the two Cu centers and is involved in hydrogen bonding interaction with the OE1 atom of Glu265. Unlike dopamine, nepicastat is not involved in a network of H-bonding though a multitude of His residues form a cage around the molecule. Conformational changes concomitant to binding can induce further interactions with one or more His residues. Furthermore, while nepicastat binds towards the opening of the active site channel, dopamine prefers binding towards the inside of the cleft. The external binding provides nepicastat with an easy access to the enzyme and leaves scope for designing analogues or addition of chemical groups that can enhance binding through more extensive hydrogen bonding or other non-covalent interaction which in turn can improve specificity and affinity of binding.

### Conclusion

The enzyme was investigated for almost three decades now that has resulted in a wealth of biochemical information. However, surprisingly, structural investigation has been rare and no three-dimensional structure is known to date for the full-length protein. The current investigation presents the first structure of DBH, albeit *in silico*, and lays the foundation for a repertoire of other investigation that would include further computational studies (viz. simulation of structures to highlight conformational changes), rational protein engineering, functional evaluation of SNPs, drug design, structure-function relationship, etc. In fact, the *in silico* model hints at why experimental structure might not have been obtained and why crystallography could be difficult. The structure seems to contain a large number of flexible loops that would inhibit the formation of diffractable crystals and would preclude good quality electron density development in the X-ray diffraction data. We also present here the first image of DBH in dimeric and tetrameric forms and the possible role of individual domains other than the catalytic domains. The C-terminal domain probably controls dimerization while the DOMON domain promotes tetramerization. This work shows that three-dimensional structure could possibly be built using biochemical information in conjunction with simple *in silico* tools and manual intervention; a new approach to structure solution. Here a subset of biochemical information has been used to orient the protein conformation, while several other biochemical data was corroborated from the structure indicating the internal consistency and accuracy of the method. The physicochemical tests corroborate the validity of the model as well. The most dramatic success has been the way the model showed why Tyr491 mutation resulted in no change in enzyme activity, a fact that eluded justification of the experimental finding for long. Similarly, it provided a structural basis to the evolutionary conservation of residue His297, which otherwise would have been difficult to understand through experimental means. The strength of the model thus lay in its ability to corroborate several experimental results. The model also provides several testable hypotheses especially with regard to the functional implications of several exonic SNPs. Site-directed mutagenesis is a simple way to test such predictions and the authors are pursuing such experimental necessities. The current investigation has resolved controversial results regarding the influence of certain SNPs on enzyme activity as well. The structure highlighted the mechanism of ligand stabilization and detailed primary structure analysis has revealed that amino acid residues responsible for intricate hydrogen bonding network and stabilization of the substrate in the catalytic site are highly conserved. Similarly, we have shown that the disulfides in the structure as well as the residues involved in interface formation are conserved across species. The conservation of interface residues argue that oligomerization of DBH subunits is important for the activity and function of the enzyme. The model paves the pathway for *in silico* drug design. For example, in case of the promising therapeutic inhibitor nepicastat, one can envisage that design of suitable inhibitor analogue or chemical modification could potentially induce multiple interactions with neighboring residues as opposed to a single hydrogen bond, which in turn can result in enhancement of specificity and strength of inhibition. The model has shown subtle differences in its catalytic site from that of rat DBH, which otherwise would have been obscure.

The model indeed suffers from some weaknesses as well. There has been high dependence on the accuracy of secondary structure predictions. It cannot still account for one disulfide bond, which could be due to the fact that in solution determination of the number of disulfides bonds was erroneous, or that the model needs further refinement and support from X-ray crystallographic studies. The authors are currently focused on the later possibility. Another intrinsic weakness includes random selection of torsion angles to fit the experimental data that may not be the most optimal possible conformation. For this, the testable hypotheses emerging from the study need to be verified experimentally. Nevertheless, the model has successfully corroborated several experimental findings and is expected to boost further investigations.

## Methods

An assortment of computational techniques, including automated homology and *ab-initio* modeling, and manual model building were used to map the three-dimensional structure of the protein. The amino acid sequence of human DBH (accession number: P09172) was obtained from NCBI (www.ncbi.nlh.nlm.gov). DBH sequences from several other species were similarly obtained. Figures were rendered using VMD or Swiss-PDB Viewer [46], [47].

### Sequence Analysis, Domain Characterization and Secondary Structure Prediction

To identify sequences similar to DBH, protein sequence search was performed using the BLAST program from NCBI available at www.ncbi.nlm.nih.gov/BLAST [48] based on an earlier report [37]. Domain analysis of sequence was accomplished using the Conserved Domain Architecture Retrieval Tool (CDART) from NCBI available at http://www.ncbi.nlm.nih.gov/Structure/lexington/lexington.cgi [38].

Computation of various physical and chemical parameters for the protein sequence was done using the ProtParam tool from ExPASy available at http://www.expasy.ch/tools/protparam [49]. DBH protein sequences known from twelve organisms including humans were aligned (multiple sequence alignment) using ClustalW program [50] available at http://align.Genome.jp. Secondary structure prediction was done using PredictProtein tool from ExPASy (http://www.predictprotein.org) [51].

### Model Building and Validation

Homology modeling tools (EsyPred3D, http://www.fundp.ac.be/sciences/biologie/urbm/bioinfo/esypred/, [52]) and threading methods (Phyre, http://www.sbg.bio.ic.ac.uk/~phyre/, [53]) of model building failed owing to lack of proper template that could provide reference coordinates for full-length protein. Instead, a combination of *ab-initio* tool and manual calculations were used to build a complete model for the enzyme structure. The protein sequence was divided into shorter amino acid stretches such that each stretch represented sequence of one or more than one domain. A total of five such amino acid stretches were considered with fragment 1 spanning the sequence from 1–56, fragment 2 from 57–173, fragment 3 from 174–195, fragment 4 from 196–526 and fragment 5 from 527–617. The polypeptide fragments were then modeled individually by *ab-initio* methods using I-TASSER server for protein structure prediction available at http://zhang.bioinformatics.ku.edu/I-TASSER [54]–[56]. For fragment 4, however, the model was obtained through homology modeling using rat PHM crystal structure (PDB ID: 1PHM) as template. To obtain the complete structure, individually modeled fragments 1, 2, 3 and 5 were assembled manually around fragment 4 with several biochemical properties of the protein as guiding milestones. The assembly of the fragments was performed using Swiss-PDB Viewer by first reproducing the ω, ψ and ϕ angles from modeled fragments to the extended polypeptide chain (617 amino acid) and then changing the torsion angles wherever necessary based on the secondary structure prediction details and location of disulfide bridges. Fragments were thus oriented to conform to available experimental data (like disulfide linkages). Close contacts between atoms and geometrical incompatibility were also considered.

After completion of assembly, energy minimization was carried out *in vacuo* with the GROMOS96 43B1 parameters, a module of Swiss-PDB Viewer, set without reaction field with different number of cycles for steepest descent and conjugate gradient runs, to relax the structure and remove any bad contacts. The model was subsequently validated using WHATIF validation server available at www.cmbi.kun.nl/WIWWWI/, [57]. Assessment of Ramachandran plot was accomplished using Rampage available at http://mordred.bioc.cam.ac.uk/~rapper/rampage.php, [58].

DBH is known to contain two Cu atoms per subunit [19], [59], [60] and the metal coordination and metal geometry has been studied in extensive details [20], [21], [28], [30]–[35]. Using these experimental data coordinates for the two Cu atoms were manually introduced into the structure. Two water molecules were also introduced around the Cu atoms conforming to experimental finding.

### Calculation of partial specific volume and circular dichroism spectrum

Partial specific volume and circular dichroism (CD) spectrum were calculated for the soluble form of the DBH model for comparison with experimental parameters [44]. Partial specific volume was calculated as ratio of protein volume to its molecular weight. Protein volume for the model was calculated using two different web servers, ^3^V server available at http://3vee.molmovdb.org/index.php [61] with 1.4 Å probe radius and VADAR available at http://vadar.wishartlab.com/ [62].CD spectrum for the model was calculated using DichroCalc available at http://comp.chem.nottingham.ac.uk/dichrocalc/ [63].

### Quaternary Structure Modeling of DBH

Subsequent to model building, the quaternary structure of the enzyme was also obtained *in silico*. It is known that the soluble enzyme tetramer is formed by non-covalent interactions between dimers [19], [27]. As such, DBH tetramer was constructed using four identical smaller subunits i.e, with the signal sequence removed (soluble form of the enzyme). It was been shown that two subunits in each tetramer are held together by two interchain disulfide bonds to form dimer [25]. The dimers were thus obtained by docking two units of monomer model obtained as above on each other using zDOCK [64] server available at http://zdock.bu.edu/ and specifying Cys528 and Cys530 as binding sites. However, zDOCK generated an output file with the binding sites facing each other but without a proper disulfide bond. The subunits in the dimer thus obtained were oriented using Swiss-PDB Viewer by rotating and translating them such that the disulfide bonds are formed with correct orientation and proximity. Several permutations and combinations were tried and the best geometrical and stereochemical fit was considered. Tetramer was subsequently modeled by docking the dimers on each other using PatchDock protein docking server available at http://bioinfo3d.cs.tau.ac.il/PatchDock [65]. The structure was further refined with FireDock (http://bioinfo3d.cs.tau.ac.il/FireDock, [66], [67].

### Tetramer Interface Identification

The non-bonded interactions in the tetramer were identified to determine the residues involved in tetramer interface formation. Hydrophobic interactions were identified by using MolProbity server available at http://molprobity.biochem.duke.edu/ [68]. First, all atom contacts were identified and screened for carbon-carbon contacts between the subunits. These C-C contacts were then considered as making hydrophobic contacts. Residues involved in electrostatic contacts were identified with a distance cut-off of <4 Å [69]. Interchain hydrogen bonds were determined using Swiss-PDB Viewer. Salt bridge identification was done using WHATIF web server available at http://swift.cmbi.ru.nl/servers/html/index.html, which considers charge interactions only over an interatomic distance less than 7 Å.

### Mapping of SNPs

The model thus obtained was analyzed to gain insight into structural characteristics. A number of exonic SNPs resulting in non-synonymous mutations in the protein, known in literature, were mapped on the three-dimensional model of DBH to understand their functional significance. Hypotheses were formulated as to their influence on enzyme activity, oligomerization and stability based on their location in the structure. Site directed mutations were obtained *in silico* using Swiss-PDB Viewer and residues within 6 Å radius of the target SNP were determined.

### Docking of Enzyme Substrate

Finally, the dopamine bound conformation of the enzyme was built manually to study the interaction of the substrate with the enzyme, in a manner similar to that reported by Prigge et al. [28] using partial model of rat DBH to determine catalytically important residues. The partial model of rat DBH (modeled using rat PHM, PDB ID: 1PHM) was compared with the human DBH model to deduce the differences in structure, if any, of the catalytic core and subsequently highlight the difference in orientation and conformation of the catalytically important residues in human DBH that are important for substrate binding.

### Docking of Inhibitor

The potentially therapeutic inhibitor nepicastat was docked on DBH model using PatchDock protein docking server available at http://bioinfo3d.cs.tau.ac.il/PatchDock. Soluble form of the enzyme was used as an input receptor structure, as it is the functional form of DBH. The three-dimensional molecular file for nepicastat was obtained from ChemSpider (http://www.chemspider.com; ChemSpider ID for nepicastat: 7971947), which was then converted into pdb format using file conversion program MN.CONVERT available at http://www.molecular-networks.com. The pdb file thus obtained was used as an input ligand structure for docking by PatchDock.

## Supporting Information

Table S1 **Respective protein lengths, domain positions and sequence identity of DBH from various organisms compared with their human counterpart.** Further details are also available in literature [37].
(DOC)

Table S2 **Residues in the model that are present in the outlier region of Ramachandran plot, their ϕ and ψ values and the type of secondary structure they belong to.**
(DOC)
