# Supplementary material for: Structural Insight of Dopamine β-Hydroxylase, a Drug Target for Complex Traits, and Functional Significance of Exonic Single Nucleotide Polymorphisms
Source: PLoS One. 2011 Oct 20;6(10):e26509. doi: 10.1371/journal.pone.0026509 (PMC3197665; doi:10.1371/journal.pone.0026509)
Supplement: Table S1 — Respective protein lengths, domain positions and sequence identity of DBH from various organisms compared with their human counterpart. Further details are also available in literature [37]. (DOC) [file pone.0026509.s001.doc]

**Table S1: Respective protein lengths, domain positions and sequence identity of DBH from various organisms compared with their human counterpart. Further details are also available in literature [37]**.

| **Organism** | **Protein length (aa)** | **DOMON domain^a^** | **Cu Type II N-terminal domain^a^** | **Cu Type II C-terminal domain^a^** | **Percent sequence similarity** |
| --- | --- | --- | --- | --- | --- |
| Homo sapiens | 617 | 57-173 | 196-344 | 359-526 | 100 |
| *Sus scrofa* | 558 | 27-142 | 166-314 | 329-495 | 86 |
| *Equus caballus* | 610 | 50-165 | 189-337 | 352-519 | 85 |
| *Bos taurus* | 610 | 50-165 | 189-337 | 352-519 | 84 |
| *Mus musculus* | 622 | 64-176 | 200-347 | 363-526 | 79 |
| *Rattus norvegicus* | 621 | 60-175 | 199-347 | 362-529 | 76 |
| *Canis lupus familiaris* | 625 | 50-163 | 189-335 | 351-516 | 74 |
| *Danio rerio* | 614 | 56-166 | 193-340 | 355-518 | 58 |
| *Drosophila melanogaster* | 670 | 104-218 | 252-393 | 413-565 | 34 |
| *Homarus americanus* | 414 | 45-184 | 188-337 | -- | 27 |
| *Aedes aegypti* | 624 | 37-151 | 202-328 | 366-492 | 24 |
| *Culex quinquefasciatus* | 676 | 35-143 | 187-321 | 368-494 | 24 |

^a^The numbers in these columns indicate start and end positions of the amino acids that define the respective domains.
