# Supplementary material for: Structural Insight of Dopamine β-Hydroxylase, a Drug Target for Complex Traits, and Functional Significance of Exonic Single Nucleotide Polymorphisms
Source: PLoS One. 2011 Oct 20;6(10):e26509. doi: 10.1371/journal.pone.0026509 (PMC3197665; doi:10.1371/journal.pone.0026509)
Supplement: Table S2 — Residues in the model that are present in the outlier region of Ramachandran plot, their ϕ and ψ values and the type of secondary structure they belong to. (DOC) [file pone.0026509.s002.doc]

**Table S2: Residues in the model that are present in the outlier region of Ramachandran plot, their φ and ψ values and the type of secondary structure they belong to.**

| **Residues** | **Phi and Psi Values** | **Secondary Structure** |
| --- | --- | --- |
| Leu30 | -62.21 and -82.84 | Helix |
| Val31 | -20.25 and -47.70 | Helix |
| Ser40 | -54.31 and 75.41 | Loop |
| Leu47 | -69.86 and -78.33 | Loop |
| Ile51 | -143.20 and 29.75 | Sheet |
| Ser58 | 60.16 and 90.43 | Loop |
| Lys82 | -46.94 and 93.70 | Loop |
| Gly107 | 7.64 and 105.95 | Loop |
| Gln119 | -28.93 and 104.23 | Loop |
| Gly121 | 172.46 and -81.65 | Loop |
| Pro127 | -68.85 and -161.81 | Loop |
| Gln128 | 66.65 and 119.64 | Loop |
| Asp155 | -38.44 and -23.08 | Loop |
| Pro156 | -84.44 and -140.66 | Loop |
| Gly164 | -58.59 and -123.64 | Loop |
| Leu173 | 165.79 and 130.02 | Loop |
| Leu180 | 120.32 and 106.99 | Loop |
| Glu181 | -166.07 and -24.91 | Loop |
| Pro200 | -119.31 and -30.57 | Loop |
| Asn201 | 2.09 and 62.10 | Loop |
| Pro208 | -104.77 and -166.44 | Loop |
| Ser225 | 125.18 and -102.84 | Loop |
| Phe279 | -66.22 and -160.27 | Loop |
| Cys283 | -31.59 and -95.92 | Loop |
| Asp290 | -64.71 and -156.45 | Loop |
| Arg291 | -91.03 and -122.51 | Loop |
| Tyr294 | -34.57 and 87.56 | Loop |
| His297 | 118.36 and 30.90 | Loop |
| Val298 | -7.65 and 147.39 | Sheet |
| Gln396 | -153.33 and -122.42 | Loop |
| Leu399 | 84.13 and -165.58 | Loop |
| Pro442 | -6.55 and 104.19 | Loop |
| Gly510 | -125.29 and 83.22 | Loop |
| Phe522 | 34.57 and -58.69 | Loop |
| Asp526 | 22.12 and 135.96 | Loop |
| Pro531 | -133.53 and 174.33 | Loop |
| Ser536 | 143.96 and -80.79 | Loop |
| Pro543 | -66.65 and -137.43 | Loop |
| Trp544 | 86.70 and -79.93 | Loop |
| Ala559 | 102.64 and 100.03 | Loop |
| Pro560 | -122.39 and -169.59 | Loop |
| His564 | 119.36 and 165.91 | Sheet |
| Cys565 | 1.20 and 92.12 | Sheet |
| Ala570 | 69.46 and 134.30 | Loop |
| Arg572 | -50.75 and -120.40 | Loop |
| Gln574 | 94.54 and 36.90 | Loop |
| Pro581 | -131.56 and -176.19 | Loop |
| Pro583 | -135.98 and -171.07 | Loop |
| Ile586 | 142.14 and -159.31 | Loop |
| Thr588 | 86.64 and 52.39 | Loop |
| Thr593 | 177.84 and -19.95 | Loop |
| Pro604 | -122.15 and 173.84 | Loop |
| Gly606 | -142.72 and 93.41 | Loop |
| Pro607 | -61.47 and 34.24 | Loop |
